# Supplementary material for: HIV-1 hijacks HGF/c-MET signaling to promote viral entry and replication in primary CD4+ T cells
Source: mBio. 2026 Oct 14;17(11):e02090-26. doi: 10.1128/mbio.02090-26 (PMC13753744; doi:10.1128/mbio.02090-26)
Supplement: Supplementary file 1 [file mbio.02090-26-s0002.pdf]

Tripathi *et al.* Supplementary figures (Fig. S1-S14) and legends

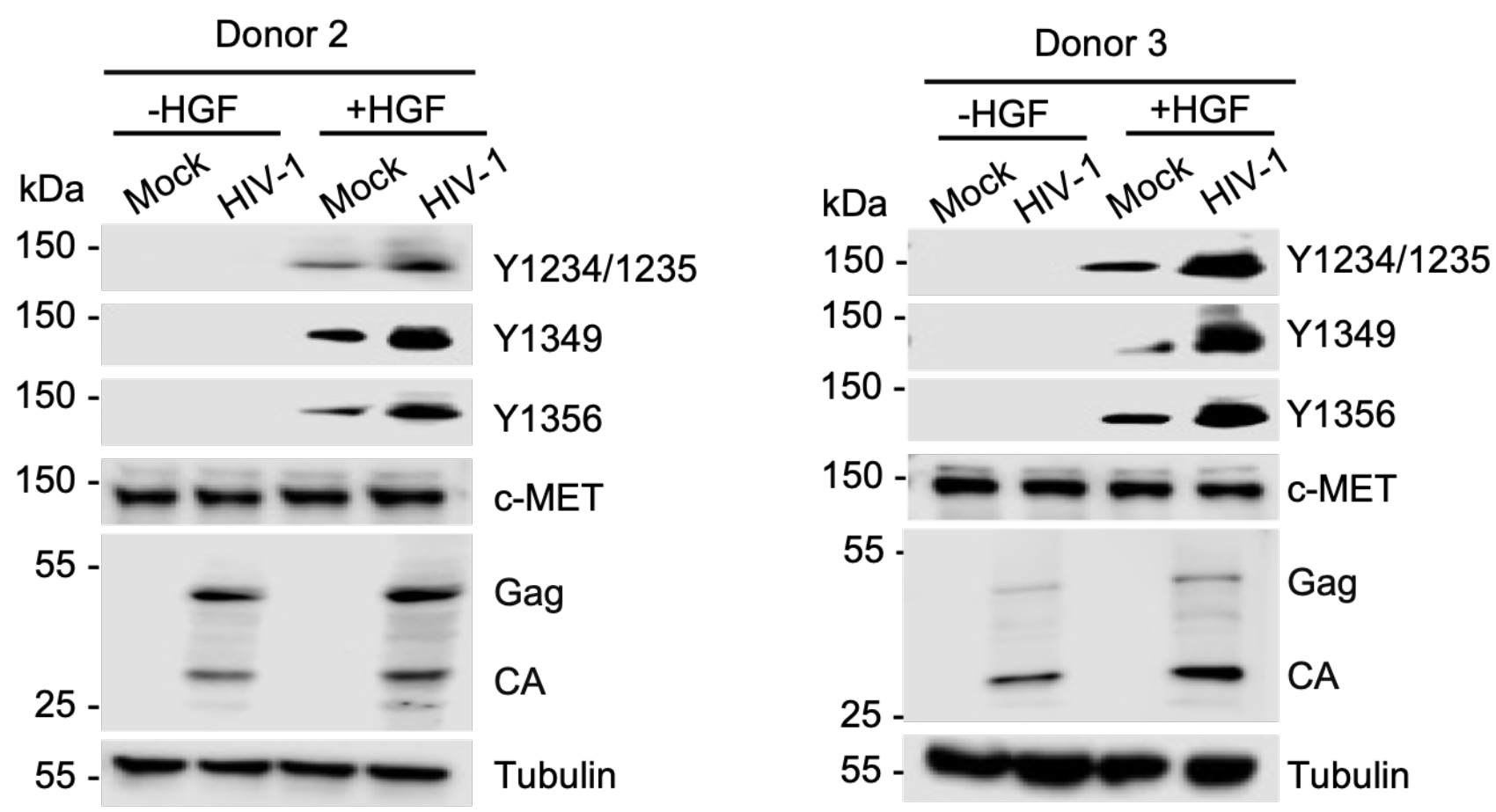

Fig. S1. HIV-1 infection promotes HGF-mediated phosphorylation of c-MET in primary CD4$^+$ T cells from donors 2 and 3. Detection of phosphorylated Y1234/1234, Y1349, and Y1356, c-MET, HIV-1 Gag and CA, and tubulin by immunoblotting. Tubulin was used as a loading control.

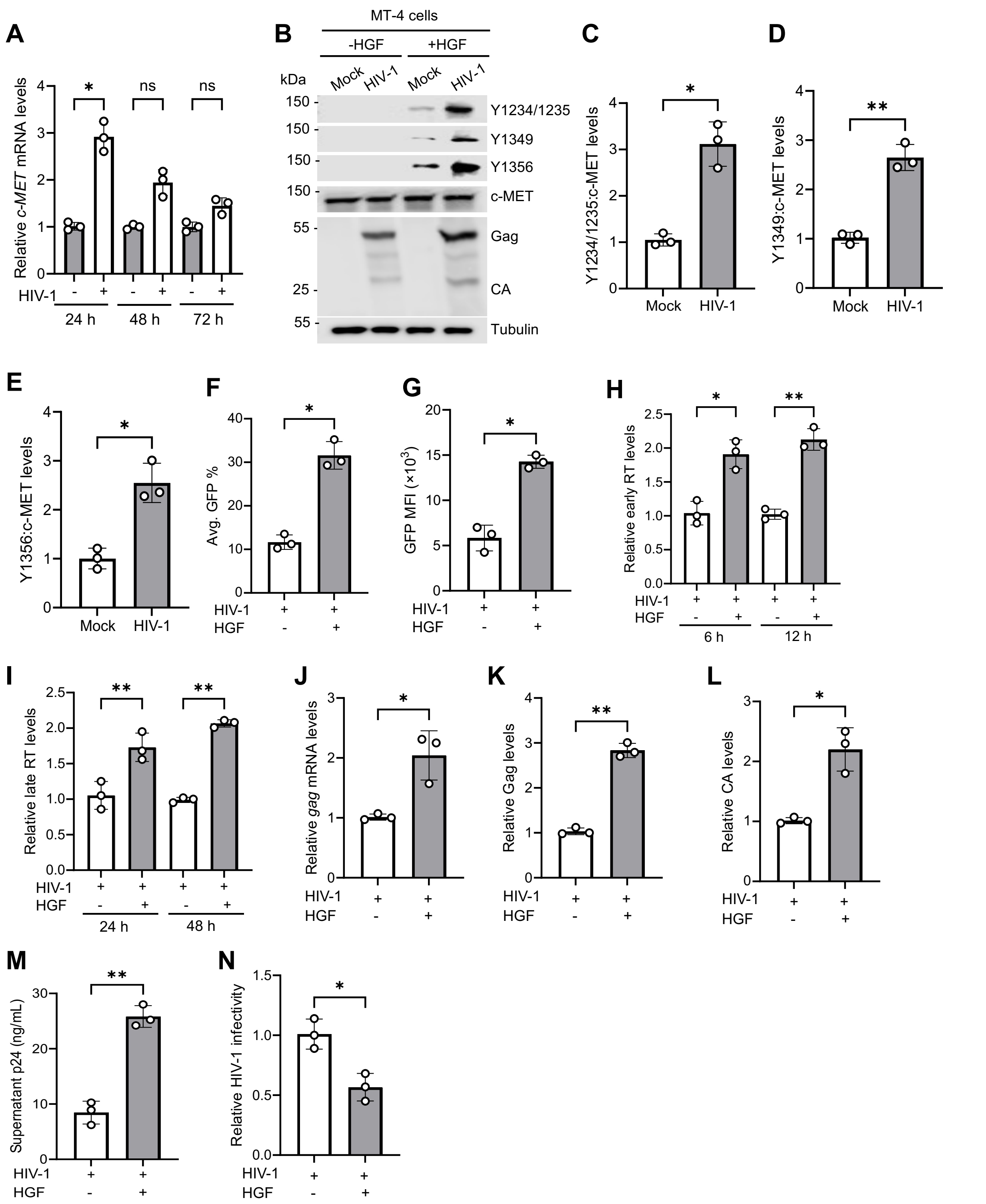

Fig. S2. HIV-1 infection promotes HGF-mediated phosphorylation of c-MET in MT-4 cells. **(A)** Time course analysis of the *c-MET* mRNA in MT4 cells at indicated time points during HIV-1 infection. **(B)** Detection of phosphorylated Y1234/1234, Y1349, and Y1356, c-MET, HIV-1 Gag

and CA, and tubulin by immunoblotting. Tubulin was used as a loading control. **(C-E)** The relative levels of phosphorylated Y1234/1235 (C), Y1349 (D), Y1356 (E) were quantified by densitometry analysis and normalized to tubulin. The level of proteins in mock-infected HGF treated cells was set to 1. **(F-G)** HIV-1 infection was measured by GFP expression using flow cytometry at 48 hpi. Changes in average GFP percentage (F) and change in MFI of GFP-positive cells (G) from HIV-1 infected MT4 cells with or without HGF treatment are shown. **(H-I)** HIV-1 early reverse transcription (early RT) products (H) and late RT products (I) were measured by qPCR at the indicated time points in HIV-1 infected MT-4 cells treated with or without HGF. Unspliced *GAPDH* was used for normalization. **(J)** Total RNA was isolated from HIV-1 infected MT-4 cells with or without HGF treatment 48 hpi, and HIV-1 *gag* mRNA levels were quantified using qRT-PCR. The amplification of *HPRT* was used as an internal control. **(K-L)** The Gag (K) and CA (L) levels were quantified by densitometry analysis and normalized to tubulin. The level of Gag and CA proteins from HIV-1-infected MT-4 cells was set to 1. **(M)** Cell culture supernatants were collected from HIV-1 infected MT-4 cells with or without HGF treatment, and p24 levels were quantified by ELISA. **(N)** TZM-bl cells were infected with the supernatant of HIV-1 infected MT-4 cells with or without HGF treatment, and luciferase activity was measured at 48 hpi. Paired T-test and one-way ANOVA multiple comparisons test were used to evaluate the statistical significance of the difference between sample groups. * $P<0.05$, ** $P<0.01$, ns, not significant.

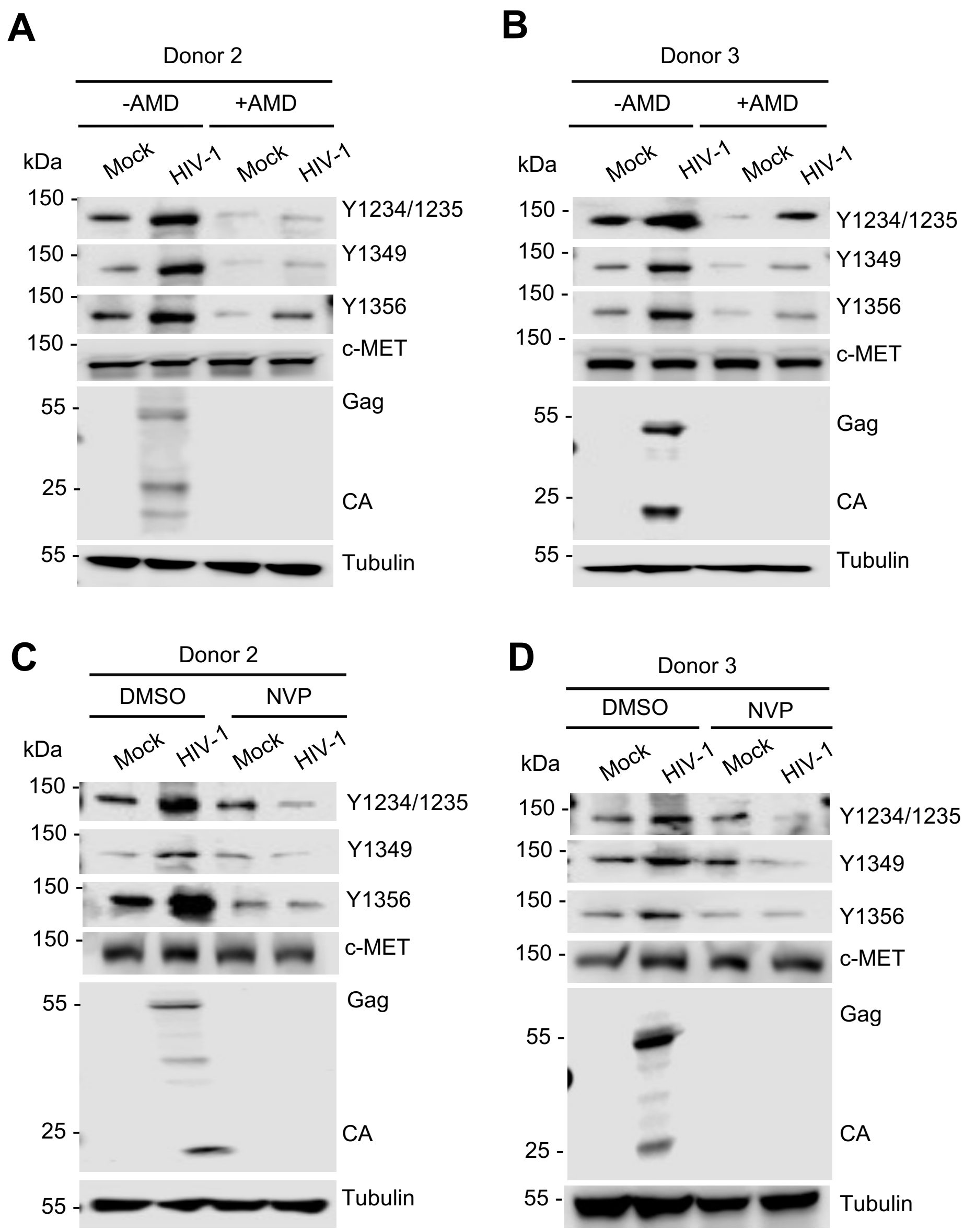

Fig. S3. AMD or NVP treatment decreases phosphorylation of c-MET levels in HGF-treated primary CD4^{+} T cells (donors 2 and 3) during HIV-1 infection. (A-D). Detection of phosphorylated Y1234/1235, Y1349, and Y1356 of c-MET, total c-MET, HIV-1 Gag, CA, and tubulin by immunoblotting in mock or HIV-1-infected primary CD4^{+} T cells treated with AMD **(A-B)**, DMSO, or NVP **(C-D)**. Tubulin was used as a loading control.

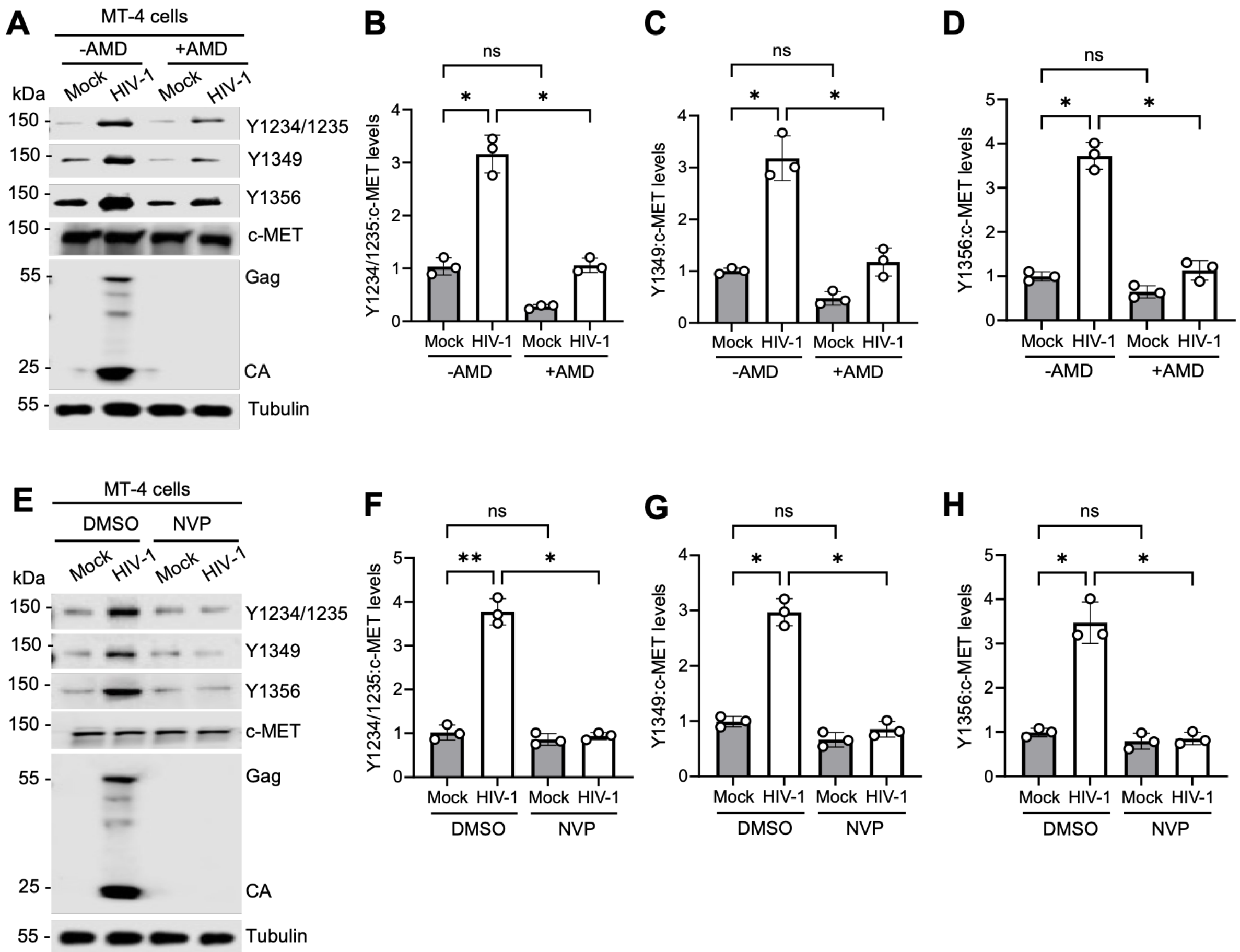

Fig. S4. AMD or NVP treatment decreases phosphorylation of c-MET levels in HGF treated MT-4 cells during HIV-1 infection. (A and **E)** Detection of phosphorylated Y1234/1235, Y1349, and Y1356 of c-MET, total c-MET, HIV-1 Gag, CA, and tubulin by immunoblotting in mock or HIV-1 infected MT-4 cells treated with AMD (A), DMSO (E), or NVP (E). Tubulin was used as a loading control. **(B-D and F-H)** The relative levels of phosphorylated Y1234/1235 (B and F), Y1349 (C and G), Y1356 (D and H) of AMD (B-D), DMSO (F-H) or NVP (F-H) treated cells were quantified by densitometry analysis and normalized to total c-MET and tubulin. The level of proteins in mock-infected DMSO treated cells was set to 1. Two-way ANOVA, Šídák's multiple comparisons test was used to evaluate the statistical significance of the difference between sample groups. * *P* <0.05, ** *P* <0.01, ns, not significant.

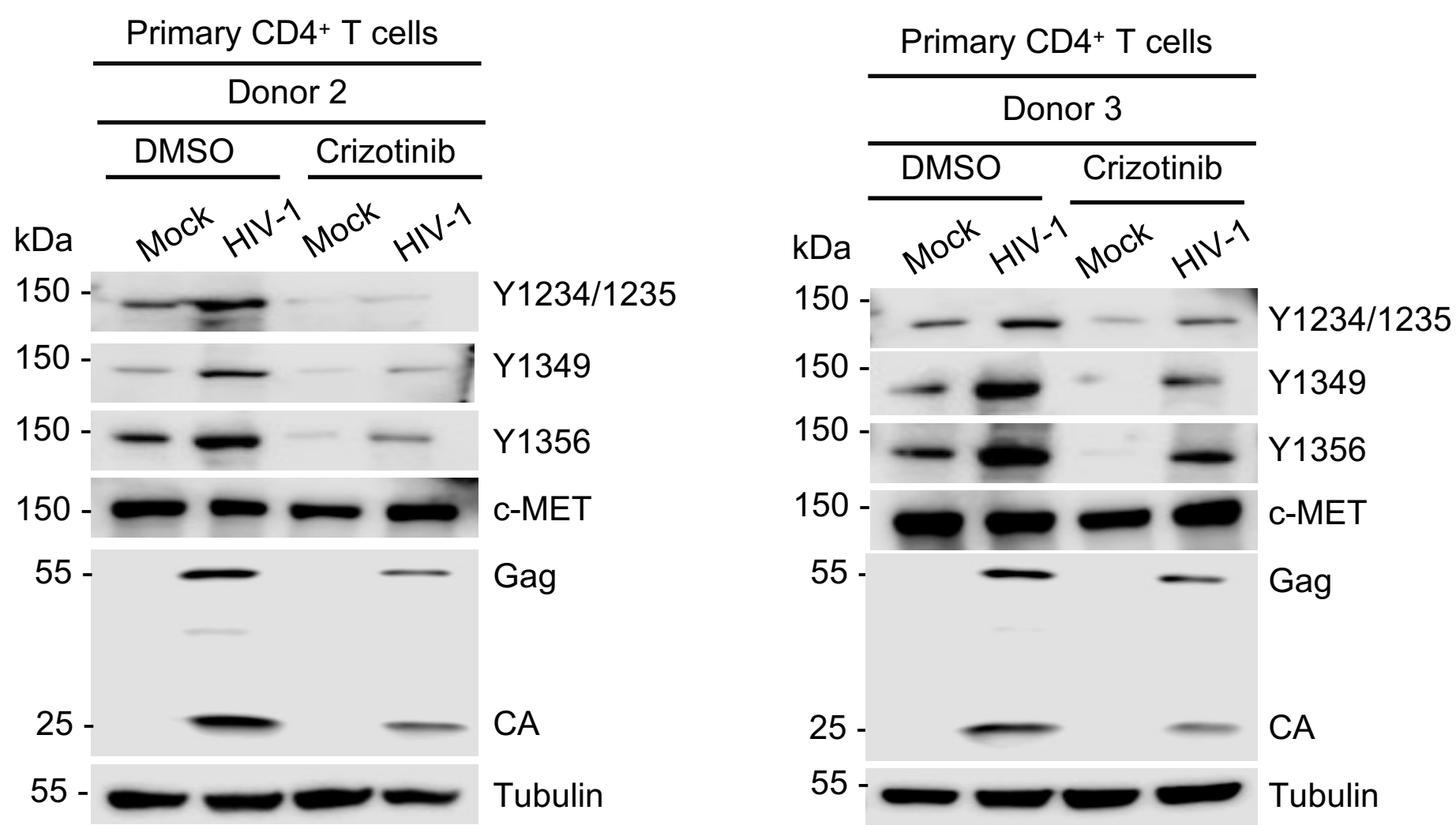

Fig. S5. Crizotinib treatment decreases HIV-1 replication in HGF-treated primary CD4$^+$ T cells from donors 2 and 3. Detection of phosphorylated Y1234/1234, Y1349, and Y1356, c-MET, HIV-1 Gag, CA, and tubulin by immunoblotting in mock or HIV-1 infected primary CD4$^+$ T cells treated with DMSO or crizotinib. Tubulin was used as a loading control.

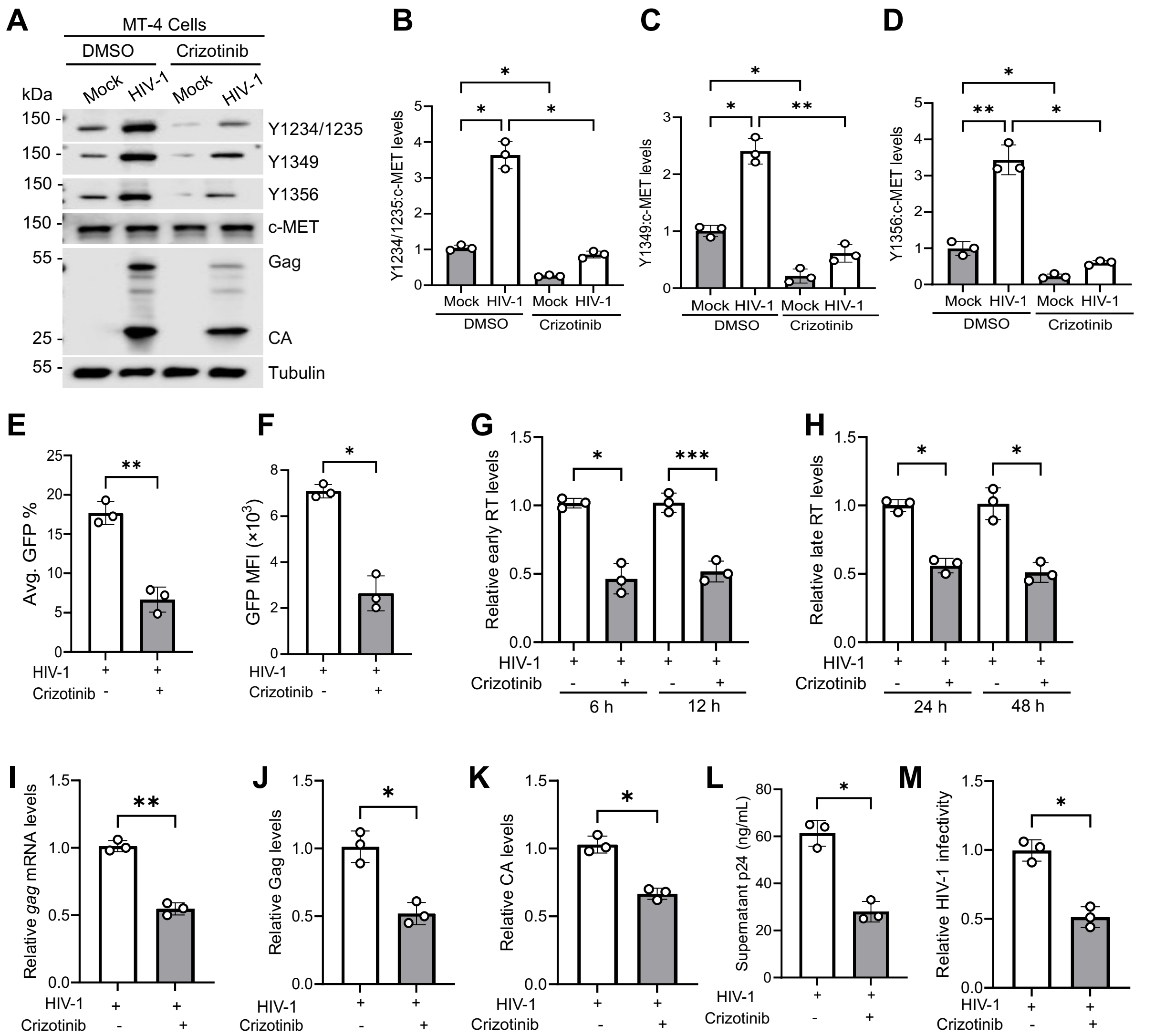

Fig. S6. Crizotinib treatment decreases HIV-1 replication and infectivity in HGF-treated MT-4 cells. (A) Detection of phosphorylated Y1234/1234, Y1349, and Y1356, c-MET, HIV-1 Gag, CA, and tubulin by immunoblotting in mock or HIV-1 infected MT-4 cells treated with DMSO or crizotinib. Tubulin was used as a loading control. **(B-D)** The relative levels of phosphorylated Y1234/1235 (B), Y1349 (C), Y1356 (D) were quantified by densitometry analysis and normalized to tubulin. The level of proteins in mock-infected HGF treated cells was set to 1. **(E-F)** HIV-1 infection was measured by GFP expression using flow cytometry at 48 hpi. Changes in average GFP percentage (E) and MFI of GFP-positive cells (F) from HIV-1 MT-4 cells treated with DMSO or crizotinib. **(G-H)** HIV-1 early RT products **(G)** and late RT products **(H)** were measured by qPCR at the indicated time points in HIV-1 infected MT-4 cells treated with DMSO or crizotinib. Unspliced *GAPDH* was used for normalization. **(I)** Total RNA was isolated from HIV-1 infected MT-4 cells

treated with DMSO or crizotinib, and HIV-1 *gag* mRNA levels were quantified using qRT-PCR. The amplification of *HPRT* was used as an internal control for RT-PCR. **(J-K)** The Gag **(J)** and CA **(K)** levels were quantified by densitometry analysis and normalized to tubulin. The level of Gag and CA proteins from mock DMSO treated cells was set to 1. **(L)** Cell culture supernatants were collected from HIV-1 infected MT-4 cells with treated with DMSO or crizotinib, and p24 levels were quantified by ELISA. **(M)** TZM-bl cells were infected with the supernatant of HIV-1 infected MT-4 cells treated with DMSO or crizotinib, and HIV-1 infectivity was measured by luciferase activities at 48 hpi. Paired T-test and one-way ANOVA multiple comparisons test were used to evaluate the statistical significance of the difference between sample groups. * $P < 0.05$, ** $P < 0.01$, *** $P < 0.001$.

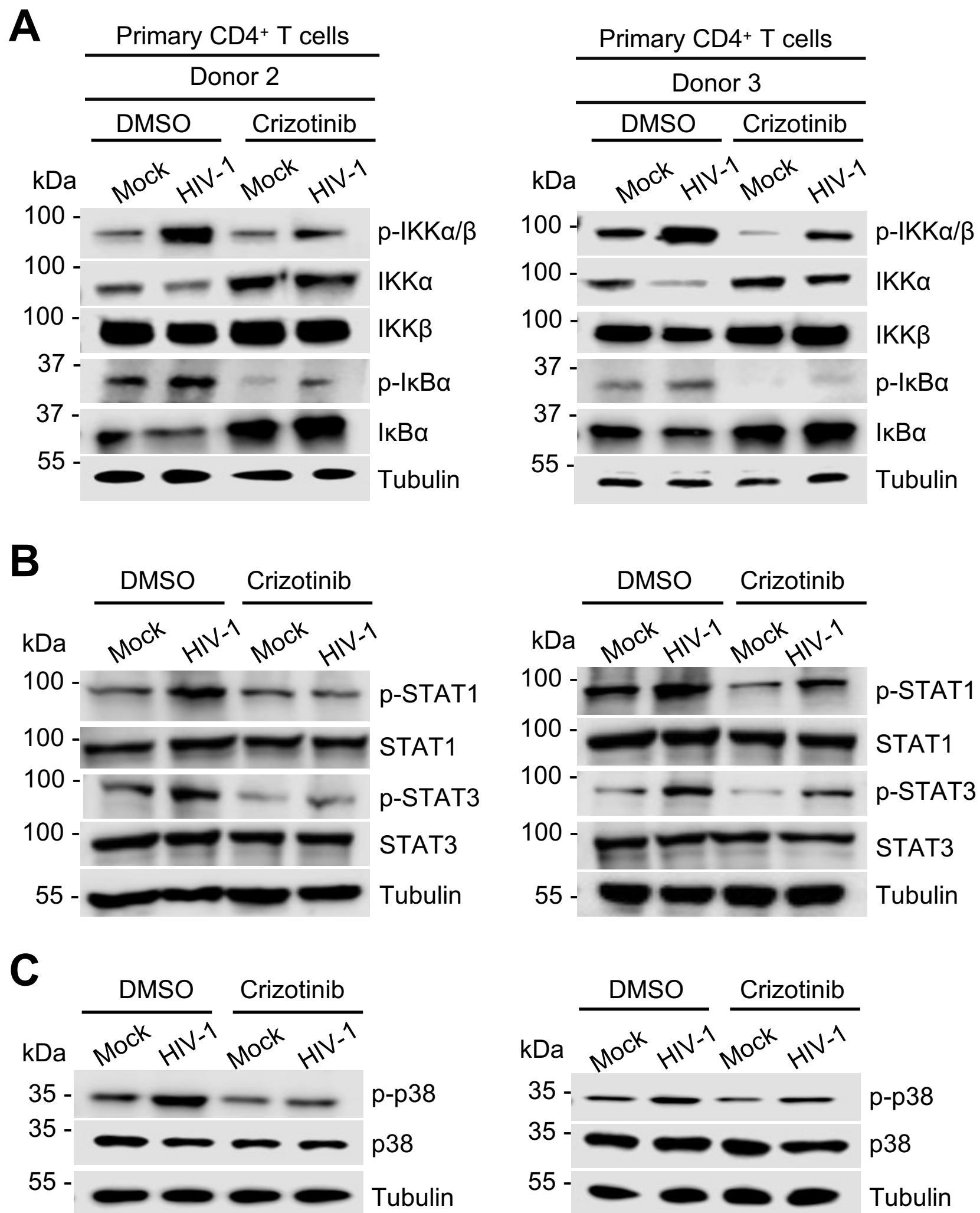

Fig. S7. Crizotinib treatment reduces p-IKKα/β, IκBα, p-STAT1, p-STAT3, and p-p38 MAPK in HGF-treated primary CD4$^+$ T cells (donors 2 and 3) during HIV-1 infection. (A-C) Mock or HIV-1 infected primary CD4$^+$ cells were treated with DMSO or crizotinib. The cell lysates were harvested and **(A)** p-IKKα/β, IKKα, IKKβ, p-IκBα, IκBα, tubulin **(B)** p-STAT1, STAT1, p-STAT3, STAT3, tubulin, and **(C)** p-p38, p38, and tubulin were detected by immunoblotting. Tubulin was a loading control.

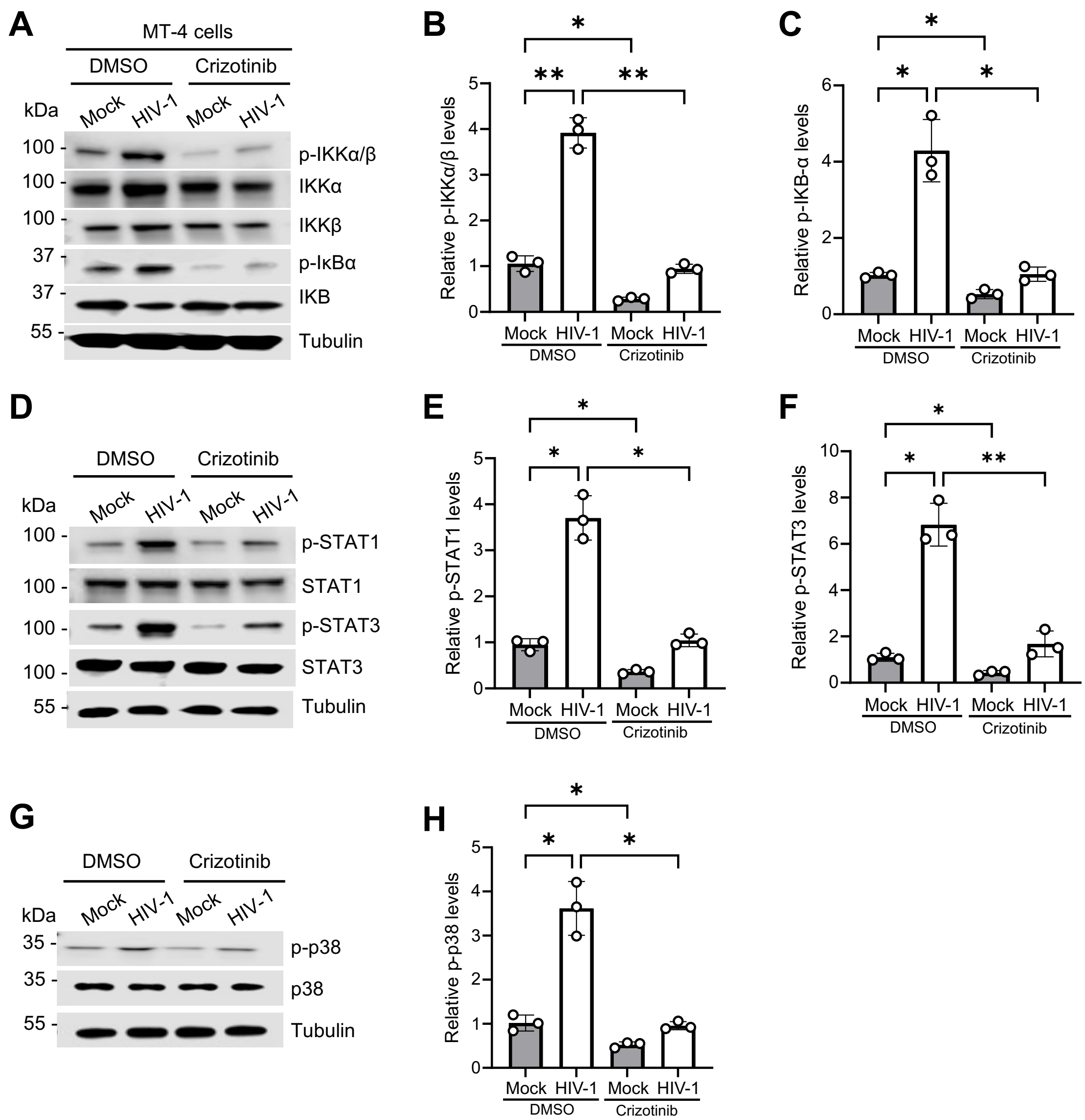

Fig. S8. Crizotinib treatment reduces p-IKKα/β, IκBα, p-STAT1, p-STAT3, and p-p38 MAPK in HGF-treated MT-4 cells during HIV-1 infection. (A, D, and **G)** Mock or HIV-1 infected MT-4 cells were treated with DMSO or crizotinib. The cell lysates were harvested and (A) p-IKKα/β, IKKα, IKKβ, p-IκBα, IκBα, and tubulin (D) p-STAT1, STAT1, p-STAT3, STAT3, tubulin and (G) p-p38, p38, and tubulin were detected by immunoblotting. Tubulin was a loading control. The relative p-IKKα/β **(B)**, p-IκBα **(C)**, p-STAT1 **(E)** p-STAT3 **(F)**, and p-p38 **(H)** levels were quantified by densitometry analysis. Relative levels were normalized to tubulin. The level of proteins in mock-infected DMSO treated cells was set to 1. Two-way ANOVA, Šídák's multiple comparisons test was used to evaluate the statistical significance of the difference between sample groups. * P <0.05, ** P <0.01.

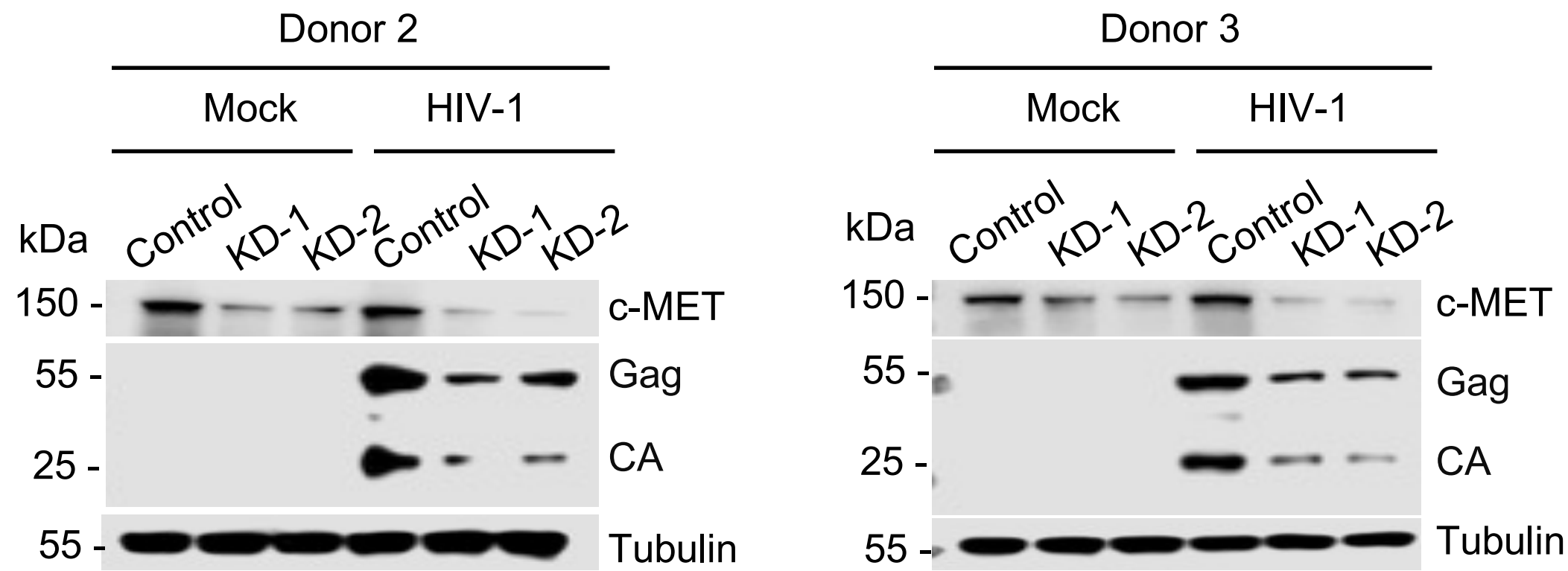

Fig. S9. c-MET KD in primary CD4⁺ T cells reduces c-MET, HIV-1 Gag and CA protein levels. KD of c-MET expression in activated primary CD4⁺ T cells from three donors using crRNP targeting two different sites in *c-MET* (KD-1 and KD-2). The cell lysates were harvested from donor 2 and 3 and the expressions of c-MET, HIV-1 Gag and CA were detected by immunoblotting. Tubulin was a loading control.

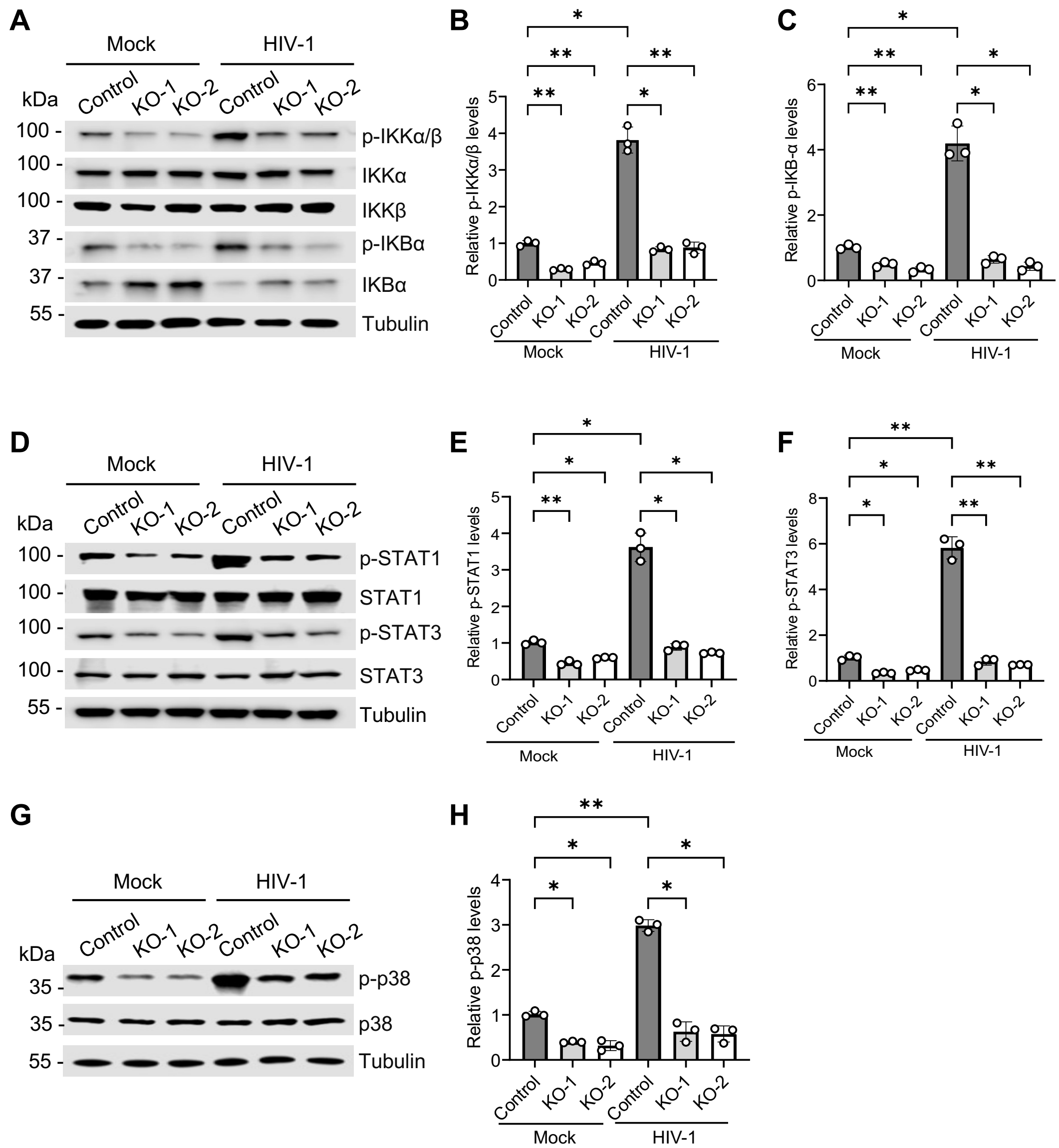

Fig. S10. c-MET KO in MT-4 cells decreases p-IKKα/β, IκBα, p-STAT1, p-STAT3, and p-p38 MAPK during HIV-1 infection. (A, D and **G)** MT-4 control and c-MET KO cells were mock or HIV-1 infected. The cell lysates were harvested and (A) p-IKKα/β, IKKα, IKKβ, p- IκBα, IκBα, and tubulin, (D) p-STAT1, STAT1, p-STAT3, STAT3 and tubulin, (G) p-p38, p-38, and tubulin were detected by immunoblotting. Tubulin was a loading control. Results from one of three independent experiments are shown. **(B, C, E, F** and **H)** Quantification results of three independent experiments. The relative p-IKKα/β (B), p-IκBα (C), p-STAT1 (E), p-STAT3 (F), p-p38 (H) levels were quantified by densitometry analysis. Relative levels were normalized to the tubulin. Two-way ANOVA, Šídák's multiple comparisons test was used to evaluate the statistical significance of the difference between sample groups. * P <0.05, ** P <0.01.

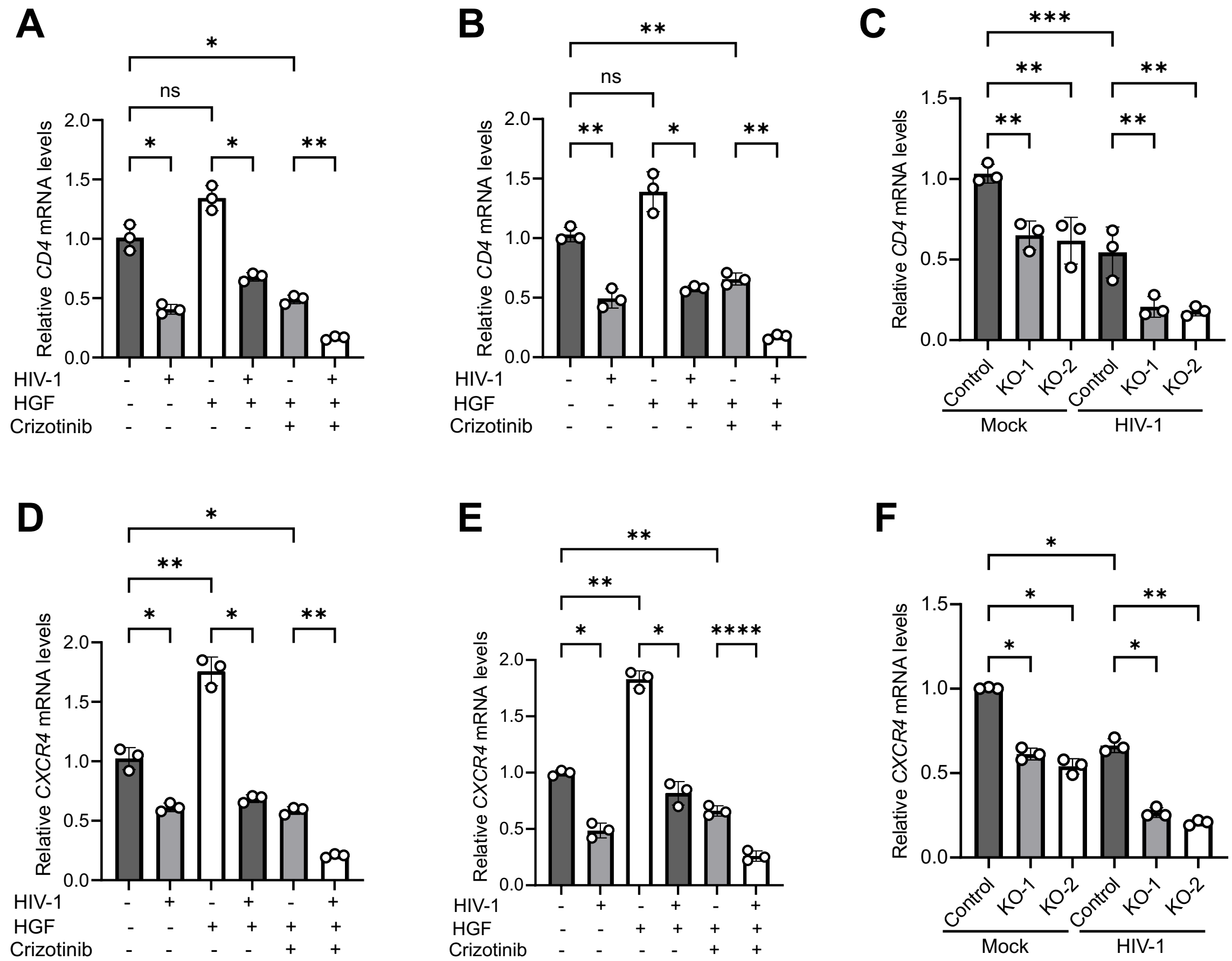

Fig. S11. Crizotinib treatment or c-MET KO reduces *CD4* and *CXCR4* transcript levels in CD4⁺ T cells. (A-D) Total RNA was isolated from mock, or HIV-1 infected three independent donor primary CD4⁺ T cells (A-B) MT-4 cells (C-D) treated either with HGF, DMSO or crizotinib, *CD4* mRNA levels (A and C) and *CXCR4* mRNA levels (B and D) were quantified using qRT-PCR. HPRT was used as an internal control. **(E-F)** Total RNA was isolated from mock or HIV-1 infected control or c-MET KO MT-4 cells, *CD4* mRNA (E) and *CXCR4* mRNA (F) levels were quantified using qRT-PCR. HPRT was used as an internal control. Two-way ANOVA, Šídák's multiple comparisons test was used to evaluate the statistical significance of the difference between sample groups. * *P* <0.05, ** *P* <0.01, *** *P* <0.001, **** *P* <0.0001, ns, not significant.

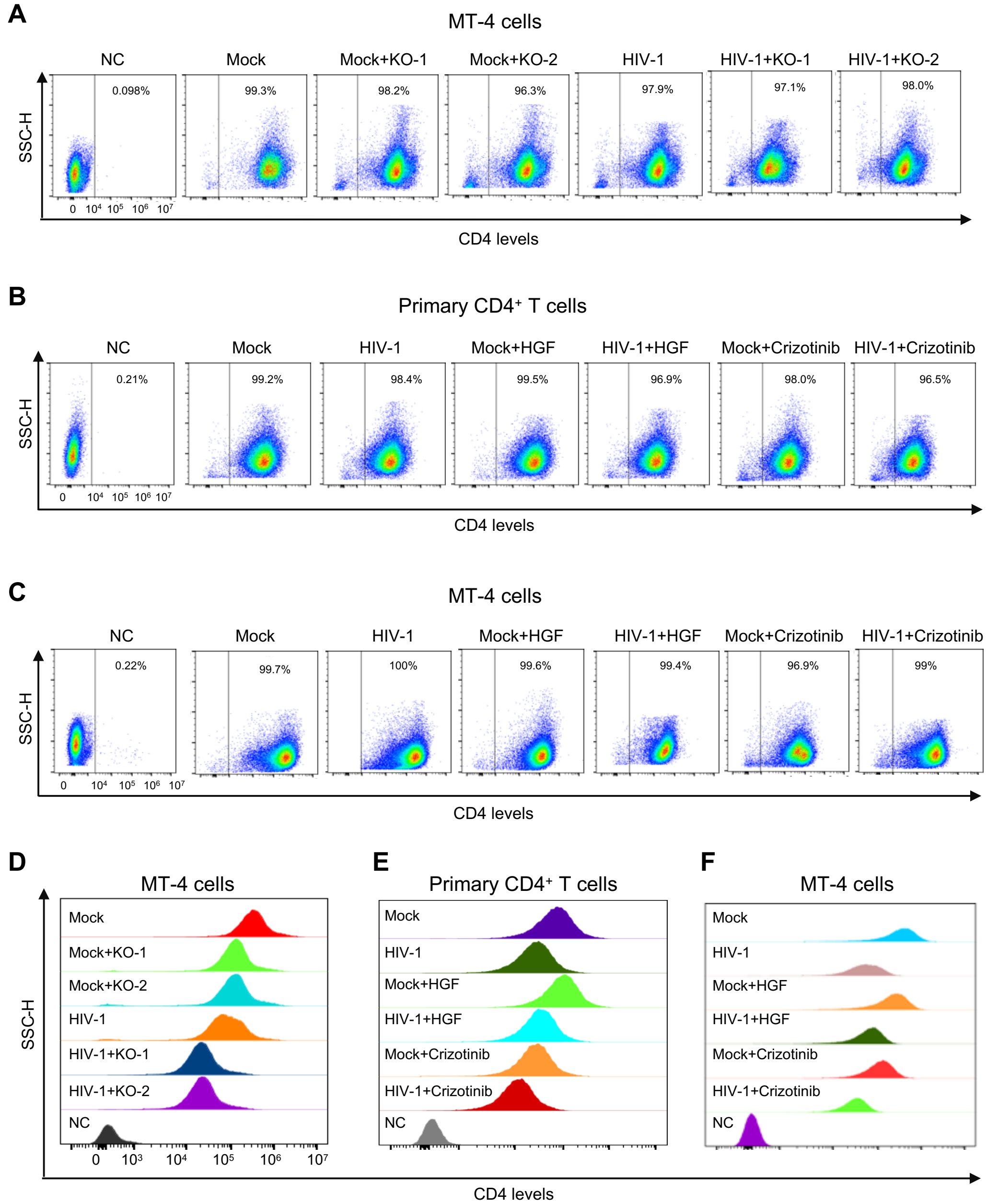

Fig. S12. Crizotinib treatment or c-MET KO reduces CD4 surface expression in CD4$^+$ T cells. (A-C) The cell surface expression of CD4 was measured by flow cytometry in mock or HIV-1 infected control and c-MET KO cells (A) mock or HIV-1-infected primary CD4$^+$ T cells (B) or MT-4 cells (C) treated with HGF or crizotinib. The percentage of CD4 positive cells are shown. **(D-F)** The histogram peaks of CD4 staining levels on control or c-MET KO MT-4 cells (D), primary CD4$^+$ T cells (E), and MT-4 cells (F) are shown. The results are representative of three independent experiments performed with biological samples (n=3) or primary CD4$^+$ T cells from 3 independent donors.

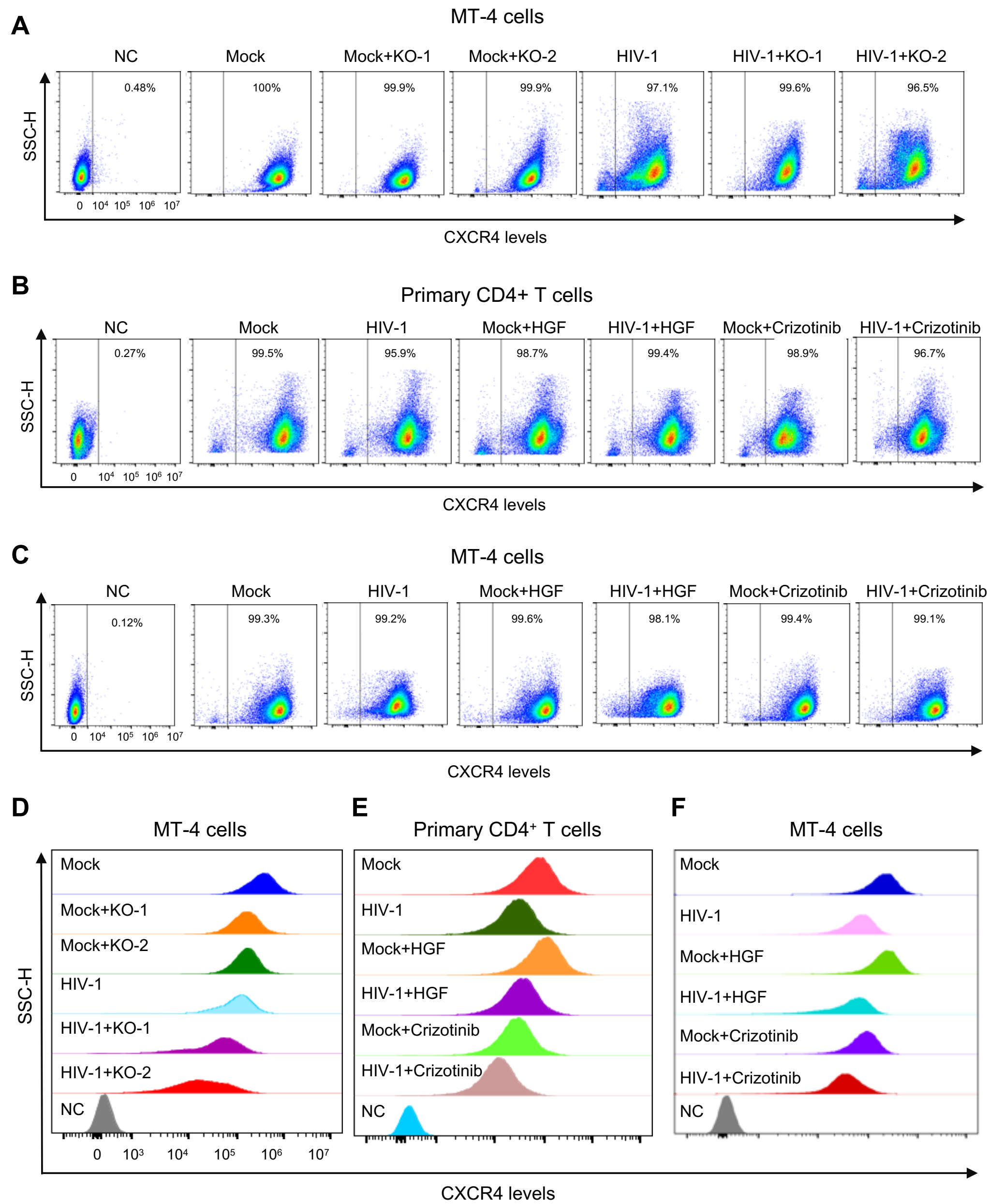

Fig. S13. Crizotinib treatment or c-MET KO reduces CXCR4 surface expression in CD4⁺ T cells. (A-C) The cell surface expression of CXCR4 was measured by flow cytometry in mock or HIV-1 infected control and c-MET KO cells (A) mock or HIV-1-infected primary CD4⁺ T cells (B) or MT-4 cells (C) treated with HGF or crizotinib. The percentage of CXCR4 positive cells are shown. **(D-F)** The histogram peaks of CXCR4 staining levels on control or c-MET KO MT-4 cells (D), primary CD4⁺ T cells (E), and MT-4 cells (F) are shown. The results are representative of three independent experiments performed with biological samples (n=3) or primary CD4⁺ T cells from 3 independent healthy donors.

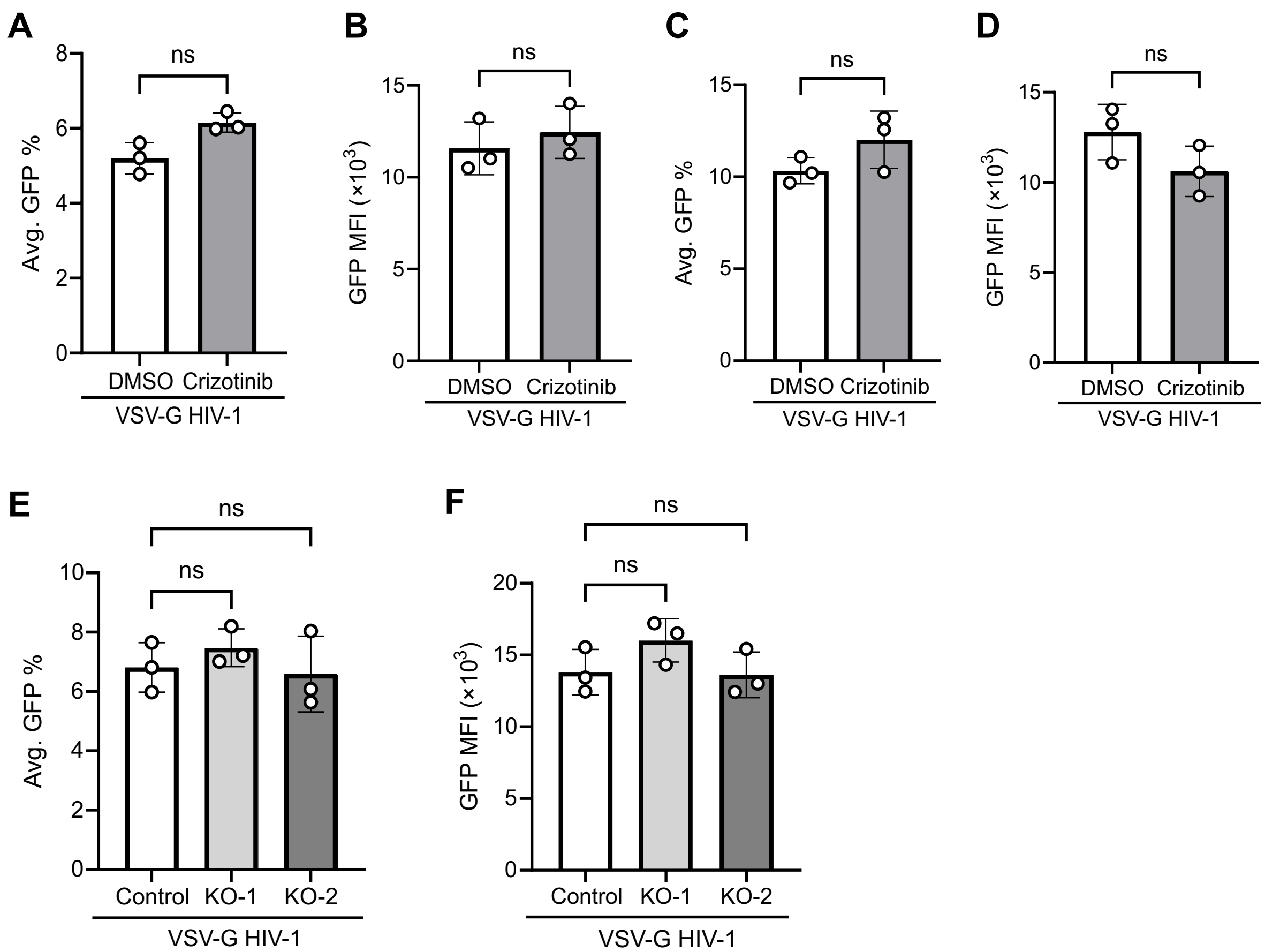

Fig. S14. Crizotinib treatment or c-MET KO does not significantly affect VSV-G-pseudotyped HIV-1 infection in CD4⁺ T cells. (A-F) VSV-G-pseudotyped HIV-1 infection was measured by GFP expression using flow cytometry at 48 hpi. Changes in average GFP percentage (A) and change in MFI of GFP-positive cells (B) from HIV-1 infected primary $CD4^+$ T cells from three independent donors upon DMSO or crizotinib treatment are shown. (C-D) Changes in average GFP percentage (C) and change in MFI of GFP-positive cells (D) from HIV-1 infected MT-4 cells upon DMSO or crizotinib treatment are shown. (E-F) Changes in average GFP percentage (E) and change in MFI of GFP-positive cells (F) from control or c-MET KO MT4 cells followed by HIV-1 infection are shown. ns, not significant.
